# Letter to the editor: Peanut butter confirmed as the source in a case of infant botulism, United Kingdom, 2024

**DOI:** 10.2807/1560-7917.ES.2025.30.40.2500713

**Published:** 2025-10-09

**Authors:** Kathleen F Gensheimer, Cecile Punzalan, Karl Klontz

**Affiliations:** 1 Epidemiology and Medical Sciences Branch, Office of Surveillance Strategy and Risk Prioritization, Human Foods Program, US Food and Drug Administration, College Park, Maryland, United States

**Keywords:** *Clostridium botulinum*, peanut butter

**To the editor: **Regarding the recent article by Crane et al. on peanut butter as a confirmed source of infant botulism, the use of the term “confirmed” is inconsistent with common practice [1]. While whole genome sequencing confirmed identical strains of *Clostridium botulinum* in the infant’s stool and in an open container of peanut butter fed to the infant, widespread presence of *C. botulinum* spores in the environment leaves open the possibility that the peanut butter container was contaminated with environmental spores. Given the infant reportedly “*had exposure to dust and soil playing in the garden*”, it is possible spores gained access to the house and open peanut butter container. No results were presented from testing unopened containers of peanut butter of the same brand and lot number that the infant consumed for *C. botulinum* spores.

*Clostridium botulinum* spores are ubiquitous and widely distributed in soil, with environmental exposure identified as an important risk factor for infant botulism [2]. Fruits, legumes, mushrooms and vegetables can be contaminated during harvesting or processing by *C. botulinum* spores that are abundant in terrestrial soils [3]. According to published scientific literature, the process of making peanut butter involves dry- or oil-roasting of shelled peanuts at temperatures and exposure times that might be insufficient to destroy all heat-resistant group I *C. botulinum* spores that are most associated with infant botulism [4]. Concerns regarding peanut butter as potentially associated with C. *botulinum* are plausible. Further, reports linking peanut butter to infant or intestinal toxaemia botulism continue to emerge in academic publications [5]. Clearly, more studies and careful investigations of infant botulism case reports are needed.

The authors did a commendable job investigating this case report. A careful food history review revealed incremental doses of a commercially prepared peanut butter fed to the infant 10 days before the onset of symptoms. An open container of peanut butter with matching *C. botulinum* suggests it was the baby’s proximal exposure. However, to support the authors’ conclusion that the peanut butter was the sole source of the potential exposure, a closed container from the same brand and lot would need to test positive and match the clinical isolate. Current policy of the United States Food and Drug Administration is to seek closed containers of any suspect food product for laboratory analysis to avoid questions regarding potential contamination within the household. Cross-contamination, especially from hygroscopic foods commonly consumed with peanut butter and frequently implicated in infant botulism cases, is conceivable and not eliminated from probability by testing an open container.

Including in the report any findings from environmental investigation, such as from testing of utensils, food preparation areas, commonly implicated foods present in the home, and any other foods consumed by the infant, would be useful for investigators of future infant botulism cases.

The open container of peanut butter was contaminated, but the question remains as to whether that contamination occurred in the home. Without more information, an ultimate source of infection cannot be confirmed. To confirm the source of infant botulism, future investigations should employ a standardised process to pursue testing of unopened containers of the same brand and lot number fed to the patients, in addition to potential cross-contamination sources. Only such a rigorous process will definitively address the source question and protect public health.
